# Patient-Reported Factors Impacting Memory and Cognition Among Women Currently or Previously Receiving Treatment for Breast Cancer

**DOI:** 10.7759/cureus.72135

**Published:** 2024-10-22

**Authors:** Heather Reens, Elizabeth Cotter, Ann C Eckardt

**Affiliations:** 1 Nursing, Molloy University, Rockville Centre, USA; 2 Psychology, New York University, New York, USA

**Keywords:** breast cancer, cognition, cognitive impairments, emq, fact cog, memory, pss, women

## Abstract

Objectives

Cognitive impairments associated with cancer and cancer treatments have yet to determine definitive causes, gold-standard assessments, or effective treatments to offset or mitigate symptoms. The purpose of the current study was to determine relationships between women with breast cancer and the negative memory impact of treatment and other factors.

Materials and methods

This online study included 171 participants receiving at least one type of treatment for breast cancer. This descriptive correlational study used two measures to assess memory and one for stress. The survey included demographics, treatments received, and memory.

Results

Results indicated increased perceived impairments among variables of age, stress, surgery, chemotherapy, radiation, and hormonal treatment modalities.

Discussion

This study established relationships between memory/cognitive impairments and the variables of age, four treatment modalities, three agents, and stress.

Conclusion

The results from this study demonstrate the importance of developing standardized assessments to identify the presence and severity of cancer and treatment-related cognitive impairments. The study will be used as a first step to developing a memory support strategy using a web-based nursing intervention considering the impact of stress.

## Introduction

Breast cancer is the most common form of cancer and the leading cause of death by any type of cancer among women, accounting for 25% of 1.68 million of the total number of cancer cases and 15% or 520,000 of cancer deaths worldwide [1]. When faced with breast cancer, women undergo physical, emotional, and mental transitions throughout their cancer trajectory [2]. They face challenges related to surgery and treatments that leave them with physical scars and missing identifications of womanhood as well as symptomology including nausea, vomiting, diarrhea, constipation, fatigue, neuropathy, and myelosuppression. Emotional and mental scarring is caused by their changing appearance, self-perception, and self-confidence. emotional, and mental transitions related to impairments in cognitive functioning among cancer patients, anecdotally called “chemo brain” or “chemo fog” are related to cancer and cancer-related treatments. This phenomenon continues to elude oncologic healthcare practitioners and confuses the patient [2]. These impairments can affect cognitive domains including memory, verbal fluency, attention, concentration, and recall [3]. Those with cognitive impairments also face a loss of control, decreased sense of self, increased stress and depression, role conflicts when returning to work, and impaired quality of life [2]. Cancer- and treatment-related cognitive impairments are estimated to affect 17% to 50% of breast cancer patients [4]. Other studies have demonstrated the presence of these cognitive impairments in up to 75% of those with non-central nervous system (CNS) cancers [5-7]. These changes do not simply diminish or reverse when treatment is completed, with these symptoms being reported in women who were treated with breast cancer up to two years after treatment in prospective longitudinal studies [8] and as long as 21 years after treatment in some cross-sectional studies [9].

While cancer and treatment-related cognitive impairments have been referenced in many studies, there still is much to learn. Cognitive decline was once thought of as a phenomenon that only occurred as a result of chemotherapy treatments. Literature has shown that upon being diagnosed with cancer, cognitive decline can begin [10], demonstrating that both the diagnosis and cancer itself can affect cognitive domain functioning. This decline furthers upon receiving treatment, not limited to chemotherapy, but has also been reported in cancer patients who have undergone surgery or received radiation, hormonal, endocrine, and targeted therapy. Some studies have indicated that cancer and treatment-related cognitive impairments continue into survivorship [10-11]. Over time, research has shown that what was once called chemo-brain or chemotherapy-related cognitive impairments has now extended to include structural changes in the brain related to cancer, the stress of the cancer diagnosis, and a full range of treatments. This phenomenon has been reported among patients with breast cancer, lymphoma, testicular cancer, and stem cell transplant recipients [4-5,7]. The combination of many factors, including the lack of a clear-cut definition, a decreased ability to identify causative mechanisms, and healthcare providers’ decreased confidence and lack of a deep understanding of the phenomenon along with an inability of current psychometric measures to assess the presence and severity of cognitive impairments in the population all point out a need for more research.

In the past, this phenomenon has been thought to be associated with fatigue, anxiety, depression, and stress associated with a cancer diagnosis [12]. Less emphasis was placed on the potential link to cancer treatment modalities, in particular, chemotherapy. As more research is being conducted, studies have found that chemotherapy can affect cognitive processes, as evidenced by changes in brain imaging; impairments have been observed in patients with malignant brain tumors and those receiving cranial surgery or brain radiation for cancer [12-13]. A lack of understanding, as it relates to the phenomenon of cancer- and treatment-related cognitive impairments among healthcare providers treating cancer patients, leads to decreased assessment and treatment [2]. Currently, no standardized assessment tools are available to identify the presence or severity of these impairments. The most widely used measurements are psychometric and neuropsychological tests, which have been developed to diagnose memory problems in various diseases and disorders. The problem with these tests is that they are not always effective for assessing this phenomenon because these cognitive impairments tend to be subtle and compensatory mechanisms may enhance patients’ performance outcomes on these tests. The purpose of the study was to determine relationships between women with breast cancer who at the time of the study were receiving or who had received at least one type of treatment and the negative impact that these treatments have or have had on their memory as reported by these participants. This study also explored if stress levels could negatively impact memory in breast cancer patients with self-reports of cognitive impairments.

## Materials and methods

Sample and setting

The sample for this study included women ages 18 to 80, diagnosed with any stage breast cancer who received at least one treatment modality for their disease. While cognitive decline has been associated with normal aging, the American Cancer Society [14] reported the median age of breast cancer diagnosis from 2012-2016 was 62 and that when measured in 10-year age groups, those in their 70s had the highest probability of being diagnosed with breast cancer. For this reason, it was important to the researchers that the sample reflected the breast cancer population at large, extending the inclusion criteria to 80 years. Women were included if they were receiving treatment at the time of the study or completed treatment no more than five years before the study.

Participants were recruited from a private practice in Long Island, NY, and through social network recruitment using snowballing methods and local and online breast cancer support groups. This was an internet-based study that used a one-time questionnaire on an online platform. This study received approval from the IRB Committee at Molloy University (Approval no. 1562703-1). Participants were informed that study participation was voluntary and could withdraw anytime without penalty. Upon accessing the web-based questionnaire, instructions for completing the survey were included. On this first page, potential participants were informed that they were consenting to participate by submitting the survey. They were also informed that they could withdraw at any time.

Study procedure

A one-time web-based Cancer and Memory PhD Study Survey designed by the researchers was provided via a recruitment flyer distributed to patients at an independent oncology office and via support groups. The recruitment flyer contained a link to the survey. Between August 26, 2020, and December 27, 2020, 217 individuals responded to the survey. The 72-item survey was divided into seven sections: an introductory question confirming they received treatment for breast cancer; demographics; types of treatments received; Perceived Stress Scale (PSS), Functional Assessment of Cancer Therapy-Cognition (FACT-Cog), Everyday Memory Questionnaire (EMQ), and one open-ended question (see Appendices) [15]. Out of the 217 initial respondents, 46 were excluded because of incomplete surveys.

This study focused on the cognitive domains of memory, cognitive abilities, and cognitive impairments. Since the use of psychometric tests has had less success in identifying cognitive impairments in patients diagnosed with cancer and receiving cancer treatments, the researchers examined participants’ perceptions of impaired memory and cognition. Memory was measured by the Perceived Cognitive Impairments (PCI) and Perceived Cognitive Abilities (PCA) subscales of the FACT-Cog and the EMQ. Stress was measured using the PSS. The questionnaire also included one open-ended question that asked participants to describe what factors they felt negatively impacted their memory. Instrument reliability was strong among the sample in this study, as demonstrated by their Cronbach’s Alpha, which can be found in Table 1.

**Table 1:** Internal reliability for instruments used in this study. FACT-Cog, functional assessment of cancer therapy-cognition.

Instrument	Analysis type	Reliability
Perceived Stress Scale (PSS)	Cronbach’s Alpha	.866 (n=167)
Everyday Memory Questionnaire (EMQ)	Cronbach’s Alpha	.934 (n=152)
FACT-Cog Perceived Cognitive Impairments (PCI) Subscale	Cronbach’s Alpha	.951 (n=165)
FACT-Cog Perceived Cognitive Abilities (PCA) Subscale	Cronbach’s Alpha	.896 (n=168)
FACT-Cog Complete Scale	Cronbach’s Alpha	.965 (n=160)

Instruments

Demographics and Sample Characteristics

Inclusion criteria for participants in this study included age between 18-80, female, English-speaking, primary diagnosis of breast cancer, currently receiving one treatment modality, or having completed therapy no more than five years before the study. Demographics assessed included age, highest level of education, race/ethnicity, and marital status. Participants were also asked about the types of breast cancer treatment modalities, agents they had or were currently receiving, and when was their last treatment.

Memory Measures

Memory was also measured using the EMQ, a 13-item Likert-type scale that subjectively measures memory failures in everyday life [16]. This measurement tool determines self-reported memory problems with questions reflecting things that may occur in daily life. Respondents are asked to reflect on memory problems within the last month. Responses on the Likert-type scale range from: A. Once or less in the last month; B. More than once a month but less than once a week; C. About once a week; D. More than once a week or less than once a day; E. Once or more in a day. Each letter represents how often the respondent has experienced each issue presented in the question. The more frequently these events occurred, the more perceived memory problems.

Cognition Measures

The FACT-Cog is a valid and reliable 37-item Likert-type scale divided into four subscales: Perceived Cognitive Abilities (PCA), Perceived Cognitive Impairment (PCI), Quality of Life, and Comments from Others [15]. For this study, it was important to the researchers that the PCA and PCI subscales were utilized as the interest for this study was looking at self-reported factors impacting memory and cognition. The FACT-Cog was used in full when determining correlations between cognition and stress. Questions on this tool focus on cognitive domains of mental acuity, concentration, memory, verbal fluency, multi-tasking/interruption, interference, and functional change. This measure has positively and negatively worded items requiring reversed scoring when analyzing data. Respondents are asked to reflect on the past seven days when answering this survey. The PIC and Comments from Others subscales are answered as never, about once a week, two to three times a week, nearly every day, and several times a day. The PCA and Impact on Quality-of-Life subscales are answered as not at all, a little bit, somewhat, quite a bit, and very much. When interpreting the results of both the PCA and PCI subscales of the FACT-Cog, the lower the scores on the scale, the more impairment in cognitive abilities and impairments.

Stress Measures

The revised 10-item Perceived Stress Scale (PSS) from Cohen and Williamson was used to assess stress in this study [17]. This measure was selected to examine stress in this sample because it is short and quick to administer, aiming to determine current stress levels through direct questions. Results on this scale are measured as “low”, “moderate”, and “high” stress.

Open-ended Question

One open-ended question in the one-time survey asked participants, “Do you feel your memory has been affected since your treatment? (Yes/No). If so, what factors do you feel have NEGATIVELY affected your memory during your breast cancer journey?” The participants were asked to be specific with their responses. This question allowed participants to narrate what they believed to have played a role in increasing their symptoms. It also allowed the researchers to examine whether the narrative responses demonstrated relationships with any quantitative results.

Data analysis

Data management, including cleaning and coding, was done using Microsoft Excel for Mac Version 16.90 (Microsoft Corp., Redmond, WA, USA). Data analysis was conducted using the statistical software program IBM SPSS Statistics for Windows, version 28 (IBM Corp., Armonk, NY, USA). Descriptive statistics for this study included age, race/ethnicity, education level, diagnosis, treatment modalities received, time since treatment completion, and stress and memory. A combination of inferential statistics was used to explore relationships among variables. Chi-square tests were used to determine categorical variables of interest and categorical outcome variables. One-way analysis of variance (ANOVA) tests with post hoc analyses were used for categorical variables of interest and continuous outcome variables. Pearson’s correlation was used to analyze relationships between continuous variables. The open-ended question at the end of the survey was analyzed by manually reading the comments to explore commonalities among participants’ responses. These were identified and clustered together into categories based on similarities.

## Results

Demographics and sample characteristics

Demographic variables assessed for this study included age, race/ethnicity, marital status, and education. Participants in this study were predominately White/Caucasian (89.5%), college-educated (49.7%), and married or partnered (76%). For the purposes of this study, all participants were female. The ages of participants in this study ranged from 24 to 80 years old (the mean age was 53.8 years). The ages of the participants in this study were broken into binned groups for analysis purposes: <49 years (n = 64, 37.65%), 50-58 years (n = 52, 30.59%), and 59 and older (n = 54, 31.76%). Study participant characteristics, including demographics, types of treatment modality (surgery, chemotherapy, radiation, hormonal therapy, and immunotherapy), and specific medications received, can be found in Table 2.

**Table 2:** Study participant characteristics. GED, General Educational Development.

Variable	N	%
Age (in years)		
< 49	64	37.4%
50-58	52	30.4%
59+	54	31.6%
Missing	1	0.6%
Race/ethnicity		
White/Caucasian	153	89.5%
Black/African American	2	1.2%
Hispanic	6	3.5%
Asian/Pacific Islander	6	3.5%
Multiple ethnicity/other	3	1.8%
Missing	1	0.6%
Education (original)		
High school graduate, diploma or equivalent (i.e., GED)	9	5.3%
Trade/technical/vocational training	7	4.1%
Some college	12	7.0%
Associate’s degree	17	9.9.%
Bachelor’s degree	68	39.8%
Graduate degree	51	29.8%
Doctorate degree	7	4.1%
Education (collapsed for smaller N)		
High school, some college, trade/technical/vocational training	28	16.4%
College graduate	85	49.7%
Graduate/doctoral degree	58	33.9%
Marital status (original)		
Single, never married	9	8.2%
Married	130	76.0%
Separated	2	1.2%
Divorced	19	11.1%
Widowed	6	3.5%
Marital status (collapsed for smaller N)		
Married or partnered	130	76.0%
Not married or partnered	41	24.0%
Treatment modalities received		
Surgery	167	97.6%
Chemotherapy	133	78.2%
Radiation	117	68.8%
Immunotherapy	35	20.5%
Hormonal therapy	130	76%
Chemotherapy agents		
Adriamycin	68	40%
Cytoxan	92	54%
Methotrexate	2	<1%
5-fluorouracil	11	<1%
Taxol	62	36%
Carboplatin	20	12%
Taxotere	58	34%
Abraxane	3	2%
Epirubicin	6	8%
Ibrance	1	1%
Xeloda	1	1%
Immunotherapy agents		
Herceptin	28	16%
Perjeta	21	12%
Kadcyla	5	3%
Verzinio	3	2%
Nerlynx	3	2%
Hormonal therapy agents		
Lupron	18	11%
Faslodex	1	1%
Tamoxifen	56	33%
Letrozole	52	31%
Anastrazole	55	32%
Aromasin	8	5%
Zoladex	9	5%

PCA and PCI

Lower scores were found on the PCA for participants who reported having undergone surgery for their breast cancer as a treatment modality (p < .05) than those who did not receive surgery. Participants who received radiation were found to have more impairments on the PCI (p = .05). Higher scores or less impairment was found to be statistically significant for those participants who reported receiving Tamoxifen than those who did not receive this medication as part of their treatment (p = .05). Regarding age, those participants who were 59 years of age and older had lower PCI scores (more impairment) than those 50-58 years (p =.01). These results can be found in Table 3.

**Table 3:** Findings of significance on PCA, PCI, EMQ, yes/no self-reported stress. PCI, Perceived Cognitive Impairments; PCA, Perceived Cognitive Abilities; EMQ, Everyday Memory Questionnaire. N, sample size; F, F-statistic or F-test; p, significance level; x2, chi-square value. * Indicates statistically significant result.

	PCA			PCI			EMQ			Yes/no stress		
	N	F	p	N	F	p	N	F	p	N	x^2^	p
Age	166	2.30	.10	167	4.51	.01*	148	4.57	.01*	170	7.51	.02*
Surgery	168	.81	.05*	169	.01	.94	150	.15	.70	113	.01	.51
Chemotherapy	167	.07	.79	168	.97	.33	149	.66	.42	112	6.13	.01*
Radiation	167	.3.21	.08	168	3.34	.05*	149	5.03	.03*	112	< .01	1.00
Hormonal therapy	168	.14	.71	169	1.35	.25	150	.55	.46	113	5.22	.02*
Adriamycin	167	.84	.36	168	.96	.33	149	4.05	.05*	113	5.30	.02*
Cytoxan	167	.29	.59	168	2.92	.09	149	6.93	.01*	113	1.55	.21
Tamoxifen	168	.57	.45	169	4.10	.05*	150	.08	.77	113	3.74	.05*

EMQ

Scores on the EMQ were found to be statistically significantly higher among those who received the chemotherapy agents Adriamycin (p =.05) and Cytoxan (p =.01), than participants who did not receive these agents meaning that they had more problems with memory. Fewer memory problems were found among those who received radiation than those who did not (p =.03). Related to age, there was a statistically significant difference between age groups (p =.01). Both the 49 years or younger and 50-58 years groups had higher EMQ (more memory problems) than those 59 years and older. These results can be found in Table 3.

Self-reports of memory problems

Self-reports of memory impairment were analyzed through Chi-square tests using the responses to the survey question, “Do you feel your memory has been affected since your treatment?” with a simple “yes” or “no” answer. Respondents were categorized into three age groups: age 49 years or less; 50-58 years; and 59 years and older. A statistically significant association was found between the three age groups included in this study and self-reports of negative effects on memory by respondents (p = .02). There were more self-reports of memory problems among those participants who received chemotherapy (p = .01) and hormonal therapy (p = .02) as part of their treatment regimen. Participants who received the chemotherapy agent Adriamycin were shown to have statistically significant differences in self-reports of memory problems with these memory problems increasing among those who did receive this medication (Pearson Chi-square, p = .01; continuity correction, p = .02). There were more self-reports of memory problems among those who received the hormonal agent, tamoxifen, than those who did not receive this drug (Pearson Chi-Square, p = .03; continuity correction, p = .05). These results can be found in Table 3.

Time since the last treatment

Time since the last treatment was assessed by asking individuals in the study when they last received any type of treatment by date or approximate date. For this analysis, results were grouped based on the year of last treatment, and items with small sample sizes were collapsed into three groups (Group 1: 1 year or less - 2020, Group 2: 1 year to 2 years - 2019, Group 3: 2 years or more - 2018 or before). All individuals participating in the study responded to this question (n= 171), with 51.46% (n= 88) responding that receiving their last treatment one year or less (within the year 2020), 17.54% (n= 30) responding that receiving their last treatment between 1 to 2 years ago (within the year 2019), and 30.99% (n= 53) responding receiving their treatment two years or more before this study (2018 or earlier).

The researchers also considered and reviewed responses from those currently taking aromatase inhibitors (AIs) for this analysis. Active treatment (i.e., surgery, chemotherapy, and radiation) was utilized in the designation of group allocation. While time did not demonstrate significance in this study, future studies may want to have clear questions regarding when treatment with surgery, chemotherapy, and radiation began and ended and when treatment with AI’s and other estrogen receptor blockers began and ended to have clearly distinguished groups for comparison. Also, a larger sample size is something to consider when analyzing the significance of the time since the last treatment.

Stress and memory using the EMQ

This study utilized both parametric and non-parametric correlation testing for the variables of stress using the PSS and memory using the EMQ. High stress was associated with more memory problems than those who reported having low or moderate levels of stress. Results from this study indicated that the higher stress levels among participants led to higher EMQ scores, indicating more memory problems. There were positive correlations between higher stress levels on the PSS and increased memory problems measured by the EMQ (Pearson correlation p < .01; Spearman’s rho, p < .01). These results can be found in Table 4.

**Table 4:** Correlations between stress and memory (EMQ). PSS, Perceived Stress Scale; PSS meaning, indicates low-, moderate-, and high-stress level); PSS raw scores, total scores of PSS not categorical level (low, moderate, or high); EMQ, Everyday Memory Questionnaire. N, sample size; F, F-statistic or F-test; p, significance level; R, correlation coefficient. * equals statistically significant difference.

Statistical results stress and memory					
Variable	N	F	p	R	p
PSS meaning and EMQ scores	145	8.29	< .01*	---	---
PSS raw scores parametric correlations	148	---	---	.373	< .01*
PSS raw scores non-parametric correlations	148	---	---	.361	< .01*

Stress and cognition using the FACT-Cog

When examining stress and cognition, this study found a statistically significant difference between the low-, moderate-, and high- -stress levels (p = < .01). When examining correlations between stress levels, participants who had moderate stress levels demonstrated fewer impairments than those with low-stress levels and those who experienced high-stress levels had fewer cognitive impairments than those experiencing moderate or low stress. Thus, higher stress levels did not necessitate a negative effect on or positive correlation with perceived cognitive impairments when using correlations between the PSS and the FACT-Cog tool for measurement. These results can be found in Table 5.

**Table 5:** Correlations between stress and cognition (FACT-Cog). PSS, Perceived Stress Scale – PSS meaning indicates low, moderate, and high stress levels; FACT-Cog, Functional Assessment of Cancer Therapy-Cognition. N, sample size; F, F-statistic or F-test; p, significance level. * Equals statistically significant difference.

Statistical results stress response and FACT-Cog			
Variable	n	F	p
PSS meaning and FACT-Cog	163	20.05	< .01*

Open-ended question

Data analysis from the open-ended responses was conducted manually, grouping them based on similarities in responses. Five categories were discovered upon analysis. The first category, treatment-related, included any comments expressing an impact on memory or cognition related to chemotherapy, surgery, hormonal therapy, or its relation to those. The category of stress/anxiety/depression included comments that were not expressed as being related to the treatment itself but instead comments naming generalized stress, anxiety, and depression. A lack of knowledge represented statements of being under or uninformed about the potential for memory and cognitive problems associated with cancer and treatments from healthcare providers and a lack of proper assessments. The struggles are in the real category, including real-life, self-reported experiences that provide examples of memory and cognitive problems. The other comments category included comments not specific enough to fit into the categories.

The treatment-related category strongly reflected quantitative results from this study, indicating that things such as chemotherapy, surgery, radiation, and hormonal therapy negatively affect memory and cognition among breast cancer patients. Stress, anxiety, and depression were related to treatments but also seen here to be independent of cancer and treatments. Of interest in this study are the reports indicating a lack of knowledge by healthcare providers about cancer- and treatment-related cognitive impairments, which leads to a decrease in education provided to cancer patients and survivors. This category also demonstrates a lack of baseline assessments or concurrent cognitive assessments throughout treatment. The struggles are real category provides a glimpse into the real-life experiences of patients who experience negative effects of memory and cognition related to cancer and treatment

## Discussion

While the phenomenon of cancer- and treatment-related cognitive impairments is not new, it is gaining more recognition in healthcare practice and research [12]. Those aiming to study cancer and treatment impairments are better understanding the multifaceted nature of occurrence, which lends itself to focusing more deeply on the various mechanisms that, when combined, can increase the occurrence of this phenomenon and the severity of the symptoms experienced. The results from this study indicate many factors, including age, certain treatment modalities, specific medications, and stress, that can negatively impact an individual’s memory or cognition.

As it relates to age in this study, younger participants had more perceived memory problems, and older participants reported more cognitive problems, reflecting that other cognitive domains may be hindered at increased levels among older patients, compared to the domain of memory. One possible explanation for this is that there are assumptions about the natural effects of aging on the domain of memory, so it could be likely that older participants felt that their memory problems were related to natural maturation and aging as opposed to the other variables in this study. Younger participants may more easily recognize memory impairments relating to executive functioning processes and work-related issues. Previous studies support this notion, exploring the negative impact of cancer- and treatment-related cognitive impairments of breast cancer patients and survivors in returning to work [18-20]. Older participants may not have represented the working population, and the lack of assessment of this variable in this study was a limitation that could be examined in future studies.

There were statistically significant differences between four treatment modalities and memory/cognition, including surgery, chemotherapy, radiation, and hormonal therapy. Memory was found to be affected in 84.8% (n = 95) of this sample. Within this group, 89.9% (n = 79) of participants who received chemotherapy reported that their memory was affected whereas individuals who did not reported 66.7% (n =16) memory affected [2]. Previous studies have supported that chemotherapy has been shown to impact memory negatively among those receiving treatment and survivors [21-26]. Participants in this study who received radiation had more self-reported cognitive impairments on the PCI, but fewer problems with memory on the EMQ than those who did not, which may reflect other factors, including treatments not assessed, may be influencing the change in cognition and memory among these participants. Another reason for this difference could be the unequal distribution of groups for those who reported receiving radiation and not in this study. For participants receiving hormonal, there were more self-reports of memory problems with “84.1% (n = 95) of this sample reporting their memory being negatively affected, and within this group, 89.3% (n = 75) who received hormonal therapy had memory problems” [2].

This study found potential relationships between two chemotherapy agents, Adriamycin and Cytoxan, and one hormonal agent, tamoxifen. All these medications were found to be associated with memory and cognitive impairments. Participants in this study who received Adriamycin had more memory impairments on the EMQ. They also had more self-reports of memory impairments than those who did not at 95.3%. Those who reported receiving Cytoxan had more memory impairments on the EMQ. Literature has documented relationships linking Adriamycin and Cytoxan, to increased memory/cognitive impairments of upwards of 70% of patients receiving these medications in combination or independently of each other [19]. Participants who received tamoxifen had fewer cognitive impairments on the PCI, but more self-reports of memory problems (94.7%) than those who did not. The literature has had varied results in establishing relationships between tamoxifen and memory and cognition problems [27,28].

This study also explored relationships between stress, memory, and cognition. Stress was characterized by levels of low, moderate, and high. Results from this study demonstrate that those with high-stress levels have more memory problems on the EMQ than those with moderate- or low-stress levels. Positive correlations were also found between stress and memory. This study investigated relationships between stress levels (low, moderate, and high) and memory using the FACT-Cog. This study found that participants who experienced high and moderate stress levels had fewer memory impairments and better quality of life than those who experienced low stress. Results here indicated that higher stress levels negatively affected memory using the PSS and EMQ. However, higher stress levels did not necessitate more perceived cognitive problems using the FACT-Cog. Previous studies have found positive correlations between cognitive decline and stress among cancer patients [29,30].

Limitations

While there was sufficient power in this study, it was a relatively small sample (n = 171). Future studies should include a larger sample size obtained from a large organization or a multi-center approach should be utilized. Diversity in the sample as it relates to race/ethnicity, education completed, and marital status was another limitation with most of the participants identifying as White/Caucasian (89.5%; n = 153), college-educated (49.7%; n = 85), and those who were married or partnered (76%; n = 130). It is recommended for future studies that questions related to treatment modalities be listed individually, with the related medication names listed under each category of modality. Breaking up these modalities and clarifying these modalities into separate questions may have benefitted participants in this study. Disease staging should also be included in future studies. Other measurements more commonly used to assess cognitive impairments may have been beneficial, potentially demonstrating more significance on other psychometric measures. This study did not examine correlations between the stage of disease and disease setting.

## Conclusions

The results of this study support the literature demonstrating the complexity of cancer- and treatment-related cognitive impairments. Relationships were found between memory/cognition and various treatment modalities, including chemotherapy, radiation, surgery, and hormonal therapy, using the FACT-Cog, EMQ, and PSS instruments. Age and medications, including Adriamycin, Cytoxan, and Tamoxifen, significantly affected memory and cognition. Previous studies utilizing psychometric tests among this population have demonstrated mixed results in determining the presence of cancer- and treatment-related cognitive impairments. For that reason, the researchers of this study decided to utilize perceived or self-reported measures. The results of the study support the need for more standardized assessments to be developed for determining the presence and severity of cancer- and treatment-related cognitive impairments in cancer patients. Without standardized assessments, problems can arise in patients reporting symptoms and healthcare providers diagnosing memory deficits. Assessments are the first step to appropriate care management plans and can assist in developing therapeutic interventions to mitigate the symptomology of this phenomenon. Developing a tool that assesses cognitive impairments and memory problems using a more subjective approach may be better at capturing these problems and thus be better at diagnosing the phenomenon. It is recommended that a tool be developed that providers could complete before initiation of therapy and at specific increments during treatment to determine if there are any changes from the patient’s baseline for future research and instrument development.

This study encourages self-reporting among cancer patients who may be suffering from alterations in memory and cognition, along with other cognitive domains. Results from this study also demonstrate the importance of healthcare provider assessment of this phenomenon among their patients. It also supports the need for increased awareness and education among healthcare providers caring for patients along their cancer trajectories. This study sheds light on the importance of future research to examine cognitive impairments to distinguish and identify probable causes and increase awareness and education among healthcare providers. This could directly benefit patients through knowledge acquisition and the development of potentially beneficial treatments. Results from this study demonstrate support for future large-scale studies reflective of a more diverse sample that includes groups with more comparable representation based on medications received. Future research on the lived experiences of cancer patients and survivors experiencing or living with memory and cognitive impairments would help support acknowledgment of these debilitating side effects that are often denoted through self-reports. More research should be conducted with a qualitative approach to determine specific problems occurring, which may help in developing a more appropriate subjective assessment tool as it may be more reflective of what cancer patients are experiencing as opposed to what researchers objectively believe are symptoms and thus attempt to measure through psychometric cognitive assessments.

## Appendices

**Table 6:** Study questionnaire [15-17]

Survey questions	Response choices
Are you now or have you completed any form of treatment for breast cancer in the last 2 years?	Yes; No
DEMOGRAPHICS The following are questions about you.	
What is your gender?	Female; Male
Birthdate	Enter Date [MM/DD/YYYY]
What is your highest level of education completed?	No schooling completed; Nursery school to 8th grade; Some high school, no diploma; High school graduate, Diploma or equivalent (i.e. GED); Some college; Trade/technical/vocational training; Associate degree; Bachelor's degree; Graduate degree; Doctorate degree
Which race/ethnicity best describes you? (Please choose only one.)	American Indian or Alaskan Native; Asian / Pacific Islander; Black or African American; Hispanic; White / Caucasian; Multiple ethnicity / Other (please specify)
What is your current marital status?	Single, never married; Married or domestic partnership; Separated; Divorced; Widowed
Cancer Treatments The following questions relate to your cancer treatments.	
What year were you initially diagnosed with breast cancer?	Enter Year [XXXX]
What treatments have you received for your breast cancer (Select all that apply)?	Surgery; Chemotherapy; Radiation; Immunotherapy; No treatment received
If you have received chemotherapy, what medications have you received? (Select all that apply).	Adriamycin (Doxorubicin); Cytoxan (Cyclophosphamide); Methotrexate; 5-FU (5-flourouracil); Taxol (Paclitaxel); Carboplatin; Taxotere (Docetaxel); Herceptin (Trastuzumab); Perjeta (Pertuzumab); Kadcyla; ENHERTU; Lupron injection (leuprolide); Faslodex injection (fulvestrant); Tamoxifen; Letrozole (femera); Anastrozole (Arimidex); N/A (not applicable); Other (please specify)
Have you completed all of your treatments for breast cancer?	Yes; No
When was the last time you received any type of treatment?	Date [MM/DD/YYYY]
What was the last type of treatment you have received?	Surgery; Chemotherapy; Radiation; Immunotherapy; No treatment received
Cancer and Memory Perceived Stress Scale (PSS) - (Cohen & Williamson, 1988)	
The questions in this scale ask you about your feelings and thoughts during the last month. In each case, you will be asked to indicate by circling how often you felt or thought a certain way. 0=Never 1=Always 2=Sometimes 3=Fairly Often 4=Very Often	
In the last month, how often have you been upset because of something that happened unexpectedly?	0 – Never; 1 – Always; 2 – Sometimes; 3 - Fairly Often; 4 - Very Often
In the last month, how often have you felt that you were unable to control the important things in your life?	0 – Never; 1 – Always; 2 – Sometimes; 3 - Fairly Often; 4 - Very Often
In the last month, how often have you felt nervous and “stressed”?	0 – Never; 1 – Always; 2 – Sometimes; 3 - Fairly Often; 4 - Very Often
In the last month, how often have you felt confident about your ability to handle your personal problems?	0 – Never; 1 – Always; 2 – Sometimes; 3 - Fairly Often; 4 - Very Often
In the last month, how often have you felt that things were going your way?	0 – Never; 1 – Always; 2 – Sometimes; 3 - Fairly Often; 4 - Very Often
In the last month, how often have you found that you could not cope with all the things that you had to do?	0 – Never; 1 – Always; 2 – Sometimes; 3 - Fairly Often; 4 - Very Often
In the last month, how often have you been able to control irritations in your life?	0 – Never; 1 – Always; 2 – Sometimes; 3 - Fairly Often; 4 - Very Often
In the last month, how often have you felt that you were on top of things?	0 – Never; 1 – Always; 2 – Sometimes; 3 - Fairly Often; 4 - Very Often
In the last month, how often have you been angered because of things that were outside of your control?	0 – Never; 1 – Always; 2 – Sometimes; 3 - Fairly Often; 4 - Very Often
In the last month, how often have you felt difficulties were piling up so high that you could not overcome them?	0 – Never; 1 – Always; 2 – Sometimes; 3 - Fairly Often; 4 - Very Often
Cancer and Memory Functional Assessment of Cancer Therapy-Cog (FACT-Cog, Ver 3)	
Below is a list of statements that other people with your condition have said are important. Please circle or mark one number per line to indicate your response as it applies to the past 7 days.	
I have had trouble forming thoughts...	0-Never; 1-About once a week; 2-Two to three times a week; 3 - Nearly everyday; 4 - Several times a day
My thinking has been slow...	0-Never; 1-About once a week; 2-Two to three times a week; 3 - Nearly everyday; 4 - Several times a day
I have had trouble concentrating...	0-Never; 1-About once a week; 2-Two to three times a week; 3 - Nearly everyday; 4 - Several times a day
I have had trouble finding my way to a familiar place...	0-Never; 1-About once a week; 2-Two to three times a week; 3 - Nearly everyday; 4 - Several times a day
I have had trouble remembering where I put things, like my keys or my wallet...	0-Never; 1-About once a week; 2-Two to three times a week; 3 - Nearly everyday; 4 - Several times a day
I have had trouble remembering new information, like phone numbers or simple instructions...	0-Never; 1-About once a week; 2-Two to three times a week; 3 - Nearly everyday; 4 - Several times a day
I have had trouble recalling the name of an object while talking to someone...	0-Never; 1-About once a week; 2-Two to three times a week; 3 - Nearly everyday; 4 - Several times a day
I have had trouble finding the right word(s) to express myself...	0-Never; 1-About once a week; 2-Two to three times a week; 3 - Nearly everyday; 4 - Several times a day
I have used the wrong word when I referred to an object...	0-Never; 1-About once a week; 2-Two to three times a week; 3 - Nearly everyday; 4 - Several times a day
I have had trouble saying what I mean in conversations with others...	0-Never; 1-About once a week; 2-Two to three times a week; 3 - Nearly everyday; 4 - Several times a day
I have walked into a room and forgotten what I meant to get or do there...	0-Never; 1-About once a week; 2-Two to three times a week; 3 - Nearly everyday; 4 - Several times a day
I have had to work really hard to pay attention or I would make a mistake...	0-Never; 1-About once a week; 2-Two to three times a week; 3 - Nearly everyday; 4 - Several times a day
I have forgotten names of people soon after being introduced...	0-Never; 1-About once a week; 2-Two to three times a week; 3 - Nearly everyday; 4 - Several times a day
My reactions in everyday situations have been slow...	0-Never; 1-About once a week; 2-Two to three times a week; 3 - Nearly everyday; 4 - Several times a day
I have had to work harder than usual to keep track of what I was doing...	0-Never; 1-About once a week; 2-Two to three times a week; 3 - Nearly everyday; 4 - Several times a day
My thinking has been slower than usual...	0-Never; 1-About once a week; 2-Two to three times a week; 3 - Nearly everyday; 4 - Several times a day
I have had to work harder than usual to express myself clearly...	0-Never; 1-About once a week; 2-Two to three times a week; 3 - Nearly everyday; 4 - Several times a day
I have had to use written lists more often than usual so I would not forget things...	0-Never; 1-About once a week; 2-Two to three times a week; 3 - Nearly everyday; 4 - Several times a day
I have trouble keeping track of what I am doing if I am interrupted...	0-Never; 1-About once a week; 2-Two to three times a week; 3 - Nearly everyday; 4 - Several times a day
I have trouble shifting back and forth between different activities that require thinking...	0-Never; 1-About once a week; 2-Two to three times a week; 3 - Nearly everyday; 4 - Several times a day
Other people have told me I seemed to have trouble remembering information...	0-Never; 1-About once a week; 2-Two to three times a week; 3 - Nearly everyday; 4 - Several times a day
Other people have told me I seemed to have trouble speaking clearly...	0-Never; 1-About once a week; 2-Two to three times a week; 3 - Nearly everyday; 4 - Several times a day
Other people have told me I seemed to have trouble thinking clearly...	0-Never; 1-About once a week; 2-Two to three times a week; 3 - Nearly everyday; 4 - Several times a day
Other people have told me I seemed confused...	0-Never; 1-About once a week; 2-Two to three times a week; 3 - Nearly everyday; 4 - Several times a day
I have been able to concentrate...	0 - Not at all; 1 - A little bit; 2 – Somewhat; 3 - Quite a bit; 4 - Very much
I have been able to bring to mind words that I wanted to use while talking to someone...	0 - Not at all; 1 - A little bit; 2 – Somewhat; 3 - Quite a bit; 4 - Very much
I have been able to remember things, like where I left my keys or wallet...	0 - Not at all; 1 - A little bit; 2 – Somewhat; 3 - Quite a bit; 4 - Very much
I have been able to remember to do things, like take medicine or buy something I needed...	0 - Not at all; 1 - A little bit; 2 – Somewhat; 3 - Quite a bit; 4 - Very much
I am able to pay attention and keep track of what I am doing without extra effort...	0 - Not at all; 1 - A little bit; 2 – Somewhat; 3 - Quite a bit; 4 - Very much
My mind is as sharp as it has always been...	0 - Not at all; 1 - A little bit; 2 – Somewhat; 3 - Quite a bit; 4 - Very much
My memory is as good as it has always been...	0 - Not at all; 1 - A little bit; 2 – Somewhat; 3 - Quite a bit; 4 - Very much
I am able to shift back and forth between two activities that require thinking...	0 - Not at all; 1 - A little bit; 2 – Somewhat; 3 - Quite a bit; 4 - Very much
I am able to keep track of what I am doing, even if I am interrupted...	0 - Not at all; 1 - A little bit; 2 – Somewhat; 3 - Quite a bit; 4 - Very much
I have been upset about these problems...	0 - Not at all; 1 - A little bit; 2 – Somewhat; 3 - Quite a bit; 4 - Very much
These problems have interfered with my ability to work...	0 - Not at all; 1 - A little bit; 2 – Somewhat; 3 - Quite a bit; 4 - Very much
These problems have interfered with my ability to do things I enjoy...	0 - Not at all; 1 - A little bit; 2 – Somewhat; 3 - Quite a bit; 4 - Very much
These problems have interfered with the quality of my life...	0 - Not at all; 1 - A little bit; 2 – Somewhat; 3 - Quite a bit; 4 - Very much
Cancer and Memory Everyday Memory Questionnaire-EMQ (Royle & Lincoln, 2009)	
Below are listed some examples of things that happen to people in everyday life. Some of them may happen frequently and some may happen very rarely. We should like to know how often on average you think each one has happened to you over the past month.	
Having to check whether you have done something that you should have done.	Once or less in the last month; More than once a month but less than once a week; About once a week; More than once a week or less than once a day; Once or more in a day
Forgetting when it was that something happened; for example, whether it was yesterday or last week.	Once or less in the last month; More than once a month but less than once a week; About once a week; More than once a week or less than once a day; Once or more in a day
Forgetting that you were told something yesterday or a few days ago, and maybe having to be reminded about it	Once or less in the last month; More than once a month but less than once a week; About once a week; More than once a week or less than once a day; Once or more in a day
Starting to read something (a book or an article in a newspaper, or a magazine) without realizing you have already read it before	Once or less in the last month; More than once a month but less than once a week; About once a week; More than once a week or less than once a day; Once or more in a day
Finding that a word is ‘on the tip of your tongue’. You know what it is but cannot quite find it.	Once or less in the last month; More than once a month but less than once a week; About once a week; More than once a week or less than once a day; Once or more in a day
Completely forgetting to do things you said you would do, and things you planned to do	Once or less in the last month; More than once a month but less than once a week; About once a week; More than once a week or less than once a day; Once or more in a day
Forgetting important details of what you did or what happened to you the day before.	Once or less in the last month; More than once a month but less than once a week; About once a week; More than once a week or less than once a day; Once or more in a day
When talking to someone, forgetting what you have just said. Maybe saying ‘what was I talking about?'	Once or less in the last month; More than once a month but less than once a week; About once a week; More than once a week or less than once a day; Once or more in a day
When reading a newspaper or magazine, being unable to follow the thread of a story; losing track of what it is about.	Once or less in the last month; More than once a month but less than once a week; About once a week; More than once a week or less than once a day; Once or more in a day
Forgetting to tell somebody something important, perhaps forgetting to pass on a message or remind someone of something.	Once or less in the last month; More than once a month but less than once a week; About once a week; More than once a week or less than once a day; Once or more in a day
Getting the details of what someone was told you mixed up and confused.	Once or less in the last month; More than once a month but less than once a week; About once a week; More than once a week or less than once a day; Once or more in a day
Forgetting where things are normally kept or looking for them in the wrong place.	Once or less in the last month; More than once a month but less than once a week; About once a week; More than once a week or less than once a day; Once or more in a day
Repeating to someone what you have just told them or asking someone the same question twice.	Once or less in the last month; More than once a month but less than once a week; About once a week; More than once a week or less than once a day; Once or more in a day
Cancer and Memory In Your Own Words	
Please answer the following question related to your own personal experience.	
Do you feel your memory has been affected since your treatment? (Yes/No).	Yes; No
If so, what factors do you feel have NEGATIVELY affected your memory during your breast cancer journey? Please be as specific as possible.	
